# High-flow nasal cannula versus noninvasive positive pressure ventilation in acute respiratory failure: interaction between PaO_2_/FiO_2_ and tidal volume

**DOI:** 10.1186/s13054-017-1861-4

**Published:** 2017-11-22

**Authors:** Yanfei Shen, Weimin Zhang

**Affiliations:** 1 Intensive care unit, Dongyang People’s Hospital, NO.60 wuning west Road, Jinhua City, Zhejiang 322100 People’s Republic of China; 2 Department of Intensive care unit, Dongyang People’s Hospital, NO.60 wuning west Road, dongyang, Jinhua, Zhejiang 322100 People’s Republic of China

We read with interest the systematic review by Zhao et al. [1] comparing the effect of the high-flow nasal cannula (HFNC) and noninvasive positive pressure ventilation (NIPPV) therapies in acute respiratory failure. Coincidentally, a similar review [2] was also published in *Chest* recently in which the cohort studies were also included. Both of these two reviews reported that, compared to HFNC, NIPPV had a similar endotracheal intubation rate in acute respiratory failure. However, the heterogeneity was significant (I2 = 53/63%).

Despite the statistical approach being appropriate, we believe this heterogeneity could be partly explained by the interactive effect between the PaO_2_/FIO_2_ and tidal volume. Simply using a random-effect model may lead to a biased conclusion.

**Fig. 1:**
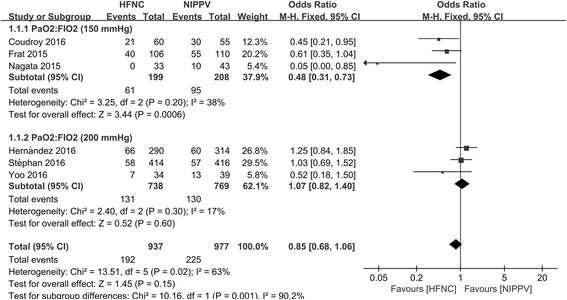
Endotracheal intubation rate (this figure is original for this article)

Six studies were included in Figure 5 of Ni et al.’s study [2]. We noticed that the mean baseline PaO_2_/FiO_2_ among these studies was largely different: in three studies (PMID 27207177, 25981908, 26106206) it was around 150 mmHg (144, 145, and 153 mmHg, respectively) and in the other three (PMID 27706464, 25980660, 26767861) it was around 200 mmHg (189, 192, and 199 mmHg, respectively). We performed a subgroup meta-analysis (Fig. 1) according to the PaO_2_/FiO_2_ levels and found that, compared to NIPPV, HFNC was associated with lower intubation rates (odds ratio (OR) 0.48; 95% confidence interval (CI) 0.31–0.73) in patients with low baseline PaO_2_/FiO_2_, while with high baseline PaO_2_/FiO_2_, the comparison was insignificant (OR 1.07; 95% CI 0.82–1.40). Besides, the heterogeneity became insignificant in these two subgroups.

The mechanism cannot be inferred from this review. Studies have indicated that lung protective ventilation during NIPPV was as important as it was during invasive mechanical ventilation in respiratory failure [3]. However, strategies such as using low tidal volumes are unlikely to work under NIPPV treatment [4] and, compared to HFNC, NIPPV was associated with higher tidal volumes, which was strongly associated with ventilator-induced lung injury.

Thus, we speculated that, in patients with low baseline PaO_2_/FiO_2_ (which to some extent indicate more severe lung injury), high tidal volume-related ventilation-induced lung injury (VILI) was more likely to happen under NIPPV therapy. Yet, both NIPPV and HFNC were still suitable for patients with mild lung injury (high PaO_2_/FiO_2_ level). This finding suggests an interactive role of PaO_2_/FiO_2_ with NIPPV and the importance of proper patient selection before NIPPV treatment. Further studies are needed to explore which acute respiratory failure patients are good candidates for NIPPV support.
